# Heritable patterns of tooth decay in the permanent dentition: principal components and factor analyses

**DOI:** 10.1186/1472-6831-12-7

**Published:** 2012-03-09

**Authors:** John R Shaffer, Eleanor Feingold, Xiaojing Wang, Karen TCuenco, Daniel E Weeks, Rebecca S DeSensi, Deborah E Polk, Steve Wendell, Robert J Weyant, Richard Crout, Daniel W McNeil, Mary L Marazita

**Affiliations:** 1 Department of Human Genetics, Graduate School of Public Health, University of Pittsburgh, Pittsburgh, PA 15261, USA; 2 Center for Oral Health Research in Appalachia, University of Pittsburgh, Pittsburgh, PA 15261, USA; 3 West Virginia University, Morgantown, WV 26506, USA; 4 Center for Craniofacial and Dental Genetics, School of Dental Medicine, University of Pittsburgh, Pittsburgh, PA 15219, USA; 5 Department of Biostatistics, Graduate School of Public Health, University of Pittsburgh, Pittsburgh, PA 15261, USA; 6 Department of Oral Biology, School of Dental Medicine, University of Pittsburgh, Pittsburgh, PA 15261, USA; 7 Department of Dental Public Health and Information Management, University of Pittsburgh, School of Dental Medicine, Pittsburgh, PA 15261, USA; 8 Department of Behavioral and Community Health Sciences, Graduate School of Public Health, University of Pittsburgh, Pittsburgh, PA 15261, USA; 9 Department of Periodontics, West Virginia University School of Dentistry, Morgantown, WV 26506, USA; 10 Dental Practice and Rural Health, West Virginia University of Dentistry, Morgantown, WV 26506, USA; 11 Clinical and Translational Science Institute, and Department of Psychiatry, School of Medicine, University of Pittsburgh, Pittsburgh, PA, USA; 12 Department of Human Genetics, Graduate School of Public Health, University of Pittsburgh, 130 DeSoto St., Pittsburgh, PA 15261, USA

**Keywords:** Dental caries genetics, Heritability, Permanent dentition, Pit and fissure surfaces, Smooth surfaces, Tooth surfaces, Principal components analysis, Factor analysis, Patterns of tooth decay, Patterns of dental caries

## Abstract

**Background:**

Dental caries is the result of a complex interplay among environmental, behavioral, and genetic factors, with distinct patterns of decay likely due to specific etiologies. Therefore, global measures of decay, such as the DMFS index, may not be optimal for identifying risk factors that manifest as specific decay patterns, especially if the risk factors such as genetic susceptibility loci have small individual effects. We used two methods to extract patterns of decay from surface-level caries data in order to generate novel phenotypes with which to explore the genetic regulation of caries.

**Methods:**

The 128 tooth surfaces of the permanent dentition were scored as carious or not by intra-oral examination for 1,068 participants aged 18 to 75 years from 664 biological families. Principal components analysis (PCA) and factor analysis (FA), two methods of identifying underlying patterns without *a priori *surface classifications, were applied to our data.

**Results:**

The three strongest caries patterns identified by PCA recaptured variation represented by DMFS index (correlation, r = 0.97), pit and fissure surface caries (r = 0.95), and smooth surface caries (r = 0.89). However, together, these three patterns explained only 37% of the variability in the data, indicating that *a priori *caries measures are insufficient for fully quantifying caries variation. In comparison, the first pattern identified by FA was strongly correlated with pit and fissure surface caries (r = 0.81), but other identified patterns, including a second pattern representing caries of the maxillary incisors, were not representative of any previously defined caries indices. Some patterns identified by PCA and FA were heritable (h^2 ^= 30-65%, p = 0.043-0.006), whereas other patterns were not, indicating both genetic and non-genetic etiologies of individual decay patterns.

**Conclusions:**

This study demonstrates the use of decay patterns as novel phenotypes to assist in understanding the multifactorial nature of dental caries.

## Background

Dental caries is a disease affecting most adults and caused by the complex interplay of numerous environmental, behavioral [1,2], and genetic risk factors [3-12]. The etiology of dental caries is further complicated by the non-uniform risk across tooth surfaces of the full dentition leading to distinct patterns of dental decay, as previously described [13-25]. Patterns of decay have been used to explore caries etiology under the assumption that different risk factors lead to distinct caries patterns. A well-known example is the maxillary anterior pattern of decay (i.e., "baby bottle" caries) in young children due in part to feeding behaviors [20,24]. Despite prevailing evidence of the importance of caries patterns, the most common indices used for studying the epidemiology of caries are DMFT and DMFS (i.e., counts of the number of decayed, missing, or filled teeth/surfaces), which do not assess specific decay patterns. As global measures of tooth decay, DMFT and DMFS indices may not be optimal for investigating genetic and environmental factors that manifest as specific patterns of caries across the dentition. Separating the global level of caries into components or patterns with distinct etiologies may be critical for identifying risk factors of modest effect sizes, such as specific genetic loci contributing to tooth decay.

Previous descriptions of caries patterns have usually assumed and compared *a priori *classifications of tooth surfaces [14-22,25], which often differed among studies, leading to inconsistencies that demonstrate the limited utility of *a priori *surface classifications. A few studies have modeled the patterns of childhood tooth decay without *a priori *assumptions and have identified distinct patterns reflecting caries of the maxillary incisor surfaces and pit and fissure surfaces, among others [13,23,24].

To our knowledge, no assessment of permanent dentition caries patterns in adults without *a priori *surface classifications has previously been performed. In this study we utilized two related analytic methodologies for identifying the underlying patterns within our dataset: principal components analysis (PCA) and factor analysis (FA). Three specific purposes of this study were (1) to identify the patterns of dental caries in the permanent dentition of adults without *a priori *assumptions about tooth surface classifications; (2) to determine the relationship between identified patterns of decay and *a priori *measures of decay such as DMFS index, decay of pit and fissure surfaces, and decay of smooth surfaces; and (3) to assess the heritability of identified patterns of decay.

## Methods

### Recruitment and data collection

The Center for Oral Health Research in Appalachia (COHRA) was created to identify the community-, family-, and individual-level predictors of oral health outcomes in the Appalachian population [26], a vulnerable subpopulation with poorer oral health compared to the greater US population [27-29]. COHRA participants were recruited by household as previously described [6,7,26], whereby eligible households were required to include at least one biological parent-offspring pair with the child being 1 to 18 years of age. All members of eligible households were invited to participate without regard to their oral health status, demography, or biological or legal relationships. Written informed consent was provided by all adult participants. Assent with parent or guardian written consent was provided on behalf of all child participants. The study was approved by the COHRA research committee and the Institutional Review Boards of the University of Pittsburgh and West Virginia University.

In total, 732 households were recruited, which comprised 2,663 individuals from 740 biological kinships of 1 to 20 family members (mean = 4.72 members). Some kinships spanned multiple households, whereas other households contained multiple kinships. Reported familial relationships were validated using panels of ancestry-informative [30] and whole-genome [31] genetic marker data provided by the Center for Inherited Disease Research at Johns Hopkins University and quality checked jointly by study investigators and the Coordinating Center for the NIH Genes and Environment Initiative (GENEVA; [32]).

Dental caries was assessed via visual inspection with a dental explorer during intra-oral dental examinations conducted by dentists or research dental hygienists calibrated with respect to a reference dentist at least once per year. Inter- and intra-examiner concordances of caries assessments were high [7,26]. Each tooth surface was scored as sound, pre-cavitated, decayed, filled, missing due to decay, or missing due to reasons other than decay, in accordance with the World Health Organization DMFS/dfs scale and in accordance with the NIH/NIDCR-approved protocol for assessing dental caries for research purposes [33]. This method of caries assessment is compatible with that recommended by the PhenX Toolkit (http://www.phenxtoolkit.org; designed to facilitate combining data across studies), and the National Center for Health Statistics Dental Examiners Procedures Manual (See Section 4.9.1.3) [34]. Third molars were excluded from caries assessment. Edentulous individuals were recruited into the study but were excluded from caries assessment and analysis.

### Statistical analysis

The analytic goal of the present study was to explore patterns of dental caries of the permanent dentition in adults. Therefore we excluded children by restricting our study sample to the 1,068 participants aged 18 to 75 years. For each participant, surface-level caries data on 128 surfaces (i.e., 4 surfaces for each incisor and canine, and 5 surfaces for each premolar and molar) were coded as 0 for sound or missing due to reasons other than decay, or coded as 1 for pre-cavitated, decayed, missing due to decay, or filled/restored. Thus, we generated a matrix of 1,068 participants by 128 indicators of surface-level caries affection status. This matrix was used as input for two related methods of extracting patterns within the data: PCA and FA [35].

PCA uses singular value decomposition of the data matrix to extract a set of uncorrelated variables (called principal components scores, PCs) where the first PC (i.e., PC1) explains the greatest possible amount of variability in the data in a single dimension, and the second PC (i.e., PC2) explains the greatest possible amount of remaining variability in the data in a single dimension orthogonal to PC1, and so on. The result is a number of orthogonal PCs equal to the number of original variables (in our data, 128), with successive PCs each explaining less and less of the data variability. Each PC can be defined as a linear combination of the original variables weighted by their loadings. The first several PCs may represent important patterns in the data, essentially assessing underlying signals from a greater number of correlated phenotype measurements. The loadings provide a way of interpreting the PCs in terms of the original variables. In other words, the loadings describe the pattern of carious lesions across the permanent dentition for a given PC, whereas the actual PCs indicate the extent/severity of caries of that decay pattern.

FA is similar to PCA in that it is used to extract latent variables called factor scores (FACs) from an original data matrix. Like PCs, FACs are calculated as linear combinations of the original variables weighted by their loadings, except that the number of FACs used to model the patterns in the data is chosen *a priori*, and the FACs are not constrained to be orthogonal. In this study, we modeled the caries data matrix using 10 factors. Like PCA, the goal of FA is to generate FACs representing underlying signals in the data matrix that can then be used as phenotypes, in this case, to identify the risk factors for dental caries.

In practice, FA and PCA often perform similarly. However the two methods take opposite perspectives in extracting patterns from a data matrix: PCA assumes that the observed variables provide the basis for the patterns, whereas FA assumes that latent patterns provide the basis for the observed variables. In this way, PCA is often used for dimension reduction, *i.e*., summarizing the information from a large number of variables with a few variables, whereas FA may better represent underlying "endophenotypes", *i.e*., unmeasured phenotypes that manifest as the observed variables. For both PCA and FA, the loadings define the patterns of decay and the PCs and FACs describe the severity of disease for their corresponding patterns.

For comparison to the PCs and FACs, we also generated three *a priori *caries phenotypes: the DMFS index, pit and fissure surface caries (PFS), and smooth surface caries (SMS). These *a priori *phenotypes are commonly used in the caries literature. DMFS was calculated as the number of pre-cavitated, decayed, missing due to decay, or filled/restored surfaces. PFS and SMS were calculated in the same way as DMFS except that counts were limited to pit and fissure surfaces and smooth surfaces, respectively. Occlusal surfaces of the premolars and molars, buccal surfaces of the maxillary molars, and lingual surfaces of the mandibular molars were considered pit and fissure surfaces. All other tooth surfaces were considered smooth surfaces.

In order to assess the stability of patterns identified by PCA and FA, we performed a sensitivity analysis by repeating PCA and FA on ten random subsets of the data comprised of 80% of the full sample. We compared the PCs and FACs obtained from random subsets to those from the full sample using the Pearson correlation coefficient, r. PCs 1-4 were extremely stable (r = 0.98 to 1.00), PCs 5-9 were stable (r = 0.86 to 0.95), and PC 10 was moderately stable (r = 0.77) across random subsets. FACs 1-6 were stable (r = 0.86 to 0.99), and FACs 7-10 were moderately stable (r = 0.69 to 0.82) across random subsets. Likewise, we assessed the effect of relatives on PCA and FA by repeating these methods in the maximal subset of unrelated individuals. PCs 1-10 and FACs 1-8 from the unrelated sample were highly correlated (r > 0.95) with those from the full sample, whereas FAC9 and FAC10 were moderately correlated (r = 0.57, and 0.81, respectively). Altogether, these results suggest that caries patterns were generally stable and robust to the inclusion of relatives among the sample.

Heritability estimates of PCs and FACs were calculated using the variance components approach. This method models phenotype correlations among all types of relatives as a function of the expected degree of genetic sharing (i.e. that parents and offspring share 50% of their genome, siblings share 50%, half-siblings share 25%, unrelated individuals share 0%, etc.). Details for this method as applied to our study sample have previously been reported [6,36]. The heritability estimate is interpreted as the proportion of phenotype variance attributable to the cumulative effect of all genes.

All statistical analyses were performed in the R software package (R Foundation for Statistical Computing, Vienna, AU), except heritability estimates which were obtained from genetic modeling performed in SOLAR [37]. Principal components analysis was performed using the *prcomp *function with default parameters. Factor analysis was performed using the *factanal *function with the Thomson's regression-based scores option, 10 factors, and other default parameters. Prevalences, correlations, and figures were all generated in R.

## Results

### Caries prevalences by surface

Surface-level caries data for 1,068 participants (ages 18 to 75 years, mean age of 34.7 years, 63.3% female, 90.0% self-reported white) across 128 tooth surfaces were collected. Tooth surfaces that exhibited evidence of pre-cavitated lesions or decay, were missing due to decay, or had been filled/restored, were considered carious. Tooth surfaces that were sound or missing due to reasons other than decay were considered non-carious. Caries prevalences per surface (i.e. the proportion of the sample exhibiting caries on a particular tooth surface) are shown in Table 1. Surfaces of the anterior maxillary teeth (i.e., incisor and canines) exhibited greater caries prevalences than anterior mandibular teeth; whereas posterior maxillary teeth (i.e., premolars and molars) exhibited lower pravelences rates than posterior mandibular teeth.

**Table 1:** Caries prevalences per surface across the permanent dentition (N = 1,068)

Surface	Right					Maxillary teeth								Left
	2	3	4	5	6	7	8	9	10	11	12	13	14	15
buccal	0.18	0.15	0.09	0.10	0.11	0.13	0.16	0.15	0.13	0.11	0.09	0.10	0.15	0.20
distal	0.18	0.21	0.20	0.18	0.07	0.11	0.16	0.16	0.13	0.08	0.19	0.21	0.20	0.18
lingual	0.22	0.40	0.08	0.07	0.07	0.15	0.15	0.16	0.16	0.08	0.07	0.09	0.39	0.23
mesial	0.16	0.27	0.19	0.12	0.07	0.15	0.17	0.18	0.16	0.09	0.12	0.20	0.25	0.18
occlusal	0.60	0.63	0.30	0.26							0.28	0.31	0.63	0.59

	right					mandibular teeth								left
	31	30	29	28	27	26	25	24	23	22	21	20	19	18

buccal	0.29	0.41	0.11	0.09	0.08	0.04	0.03	0.03	0.03	0.07	0.09	0.10	0.39	0.28
distal	0.16	0.26	0.19	0.08	0.02	0.03	0.03	0.02	0.03	0.02	0.08	0.18	0.26	0.15
lingual	0.16	0.19	0.07	0.02	0.01	0.01	0.02	0.01	0.02	0.02	0.03	0.07	0.21	0.14
mesial	0.22	0.25	0.11	0.05	0.03	0.02	0.02	0.03	0.02	0.03	0.04	0.10	0.25	0.21
occlusal	0.64	0.60	0.27	0.12							0.14	0.26	0.59	0.61

### Principal components analysis

**Figure 1:**
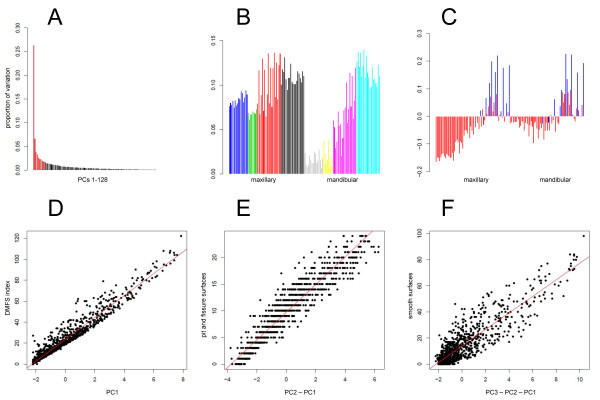
**Principal component analysis**. (A) proportion of data variance explained by PCs 1-10 (red) and successive PCs (black). (B) Loadings for PC1 ordered by tooth type, from left to right: maxillary incisors (blue), canines (green), premolars (red), molars (black), mandibular incisors (gray), canines (yellow), premolars (magenta), molars (cyan). For each tooth, contributions of surfaces are listed in the following order: buccal, distal, lingual, mesial, and occlusal, if applicable. Teeth ordered from left to right: maxillary 8, 9, 7, 10, 6, 11, 5, 12, 4, 13, 3, 14, 2, 15; mandibular 24, 25, 23, 26, 22, 27, 21, 28, 20, 29, 19, 30, 18, 31. (C) Loadings for PC2, in the same order; smooth surfaces shaded red, pit and fissure surfaces shaded blue. Scatter plots of (D) PC1 vs. DMFS index, (E) PC2 vs. pit and fissure caries, (F) PC3 vs. smooth surface caries.

PCA was performed on the surface-level data in order to extract the underlying patterns of caries. PC1 explained 26.3% of the variability in the data, PC2 explained 6.7%, and all other PCs explained < 5% (Figure 1A). Loadings show that except for anterior mandibular surfaces, all other surfaces contribute similarly to PC1 (Figure 1B) representing a near-global pattern/extent of decay. Loadings for PC2 show opposite contributions of smooth surfaces and pit and fissure surfaces (Figure 1C). Loadings for PC3 show opposite contributions of premolar vs. other surfaces and loadings for PC4 show opposite contributions of maxillary vs. mandibular surfaces (see Additional file 1). Loadings for all other PCs show complex patterns of contributions from tooth surfaces that are not easily discernible in the context of PCs 1 to 4, however, general descriptions of the contributing surfaces are summarized in Table 2.

**Table 2:** General interpretations of PCA and FA loadings.

Pattern	General interpretation of loadings
PCA	
PC1	all maxillary teeth and mandibular premolars and molars
PC2	molars vs. non-molars
PC3	premolars vs. non-premolars
PC4	mandibular teeth vs. maxillary teeth
PC5	2^nd ^molars vs. mandibular 1^st ^molars
PC6	mandibular premolars and 2^nd ^molars vs. mandibular 1^st ^molar and maxillary molars and 2^nd ^premolar
PC7	maxillary premolars and mandibular molars vs. maxillary molars and mandibular premolars
PC8	complex contributions
PC9	complex contributions
PC10	right vs. left mandibular molars
FA	
FAC1	posterior teeth: premolars and molars
FAC2	maxillary anterior teeth: incisors and canines
FAC3	mandibular canines and premolars
FAC4	maxillary premolars
FAC5	mandibular incisors and canines
FAC6	non-occlusal premolar and molar surfaces, maxillary lateral incisors, and maxillary canines
FAC7	tooth 20 (left mandibular 2^nd ^premolar)
FAC8	tooth 29 (right mandibular 2^nd ^premolar)
FAC9	maxillary 2^nd ^molars
FAC10	tooth 13 (left maxillary 2^nd ^premolar)

See Additional file 1 for full details

PC1 was nearly identical to DMFS index (r = 0.969; p-value < 10^-250 ^[i.e., the minimum p-value reported using the statistics software]; Figure 1D) indicating that the strongest pattern of caries in the data distinguished individuals by global level of decay. PFS, the count of carious pit and fissure surfaces, was very highly correlated with PC2 after subtracting out PC1 (r = 0.947; p-value < 10^-250^; Figure 1E). SMS, the count of carious smooth surfaces, was highly correlated with PC3 after subtracting out PC2 and PC1 (r = 0.894; p-value < 10^-250^; Figure 1E). These correlations show that PC1, PC2, and PC3 capture the patterns of dental decay corresponding to *a priori *phenotypes, DMFS index, excess PFS (for a given DFMS), and excess SMS (for given DMFS and PFS), respectively.

The heritability (h^2^) of DMFS index and PCs 1-10 were calculated while simultaneously adjusting for the effects of age, age^2^, and sex (Table 3). DMFS index, PC1, PC5, and PC7 were all strongly heritable (h^2 ^= 37% to 50%; p-values = 0.043 to 0.008) indicating that some patterns of dental decay were due to genetic etiologies. Other PCs were not heritable indicating that some patterns of dental decay were not due to genetics. Covariates age, age^2^, and sex explained about 10% of variation in PC1 and very little variation for the remaining PCs.

**Table 3:** Heritability estimates for DMFS index, PCs, and FACs

Phenotype	h^2^	h^2 ^SE	p-value	R^2^
DMFS	0.418	0.164	0.008	0.054
PCA				
PC1	0.404	0.160	0.009	0.095
PC2	0.149	0.171	0.190	0.017
PC3	0.000	-	0.500	0.037
PC4	0.174	0.234	0.231	0.004
PC5	0.373	0.207	0.043	0.021
PC6	0.027	0.236	0.455	0.001
PC7	0.503	0.221	0.020	0.004
PC8	0.000	-	0.500	0.003
PC9	0.000	-	0.500	0.006
PC10	0.000	-	0.500	0.001
FA				
FAC1	0.157	0.181	0.194	0.033
FAC2	0.000	-	0.500	0.014
FAC3	0.653	0.198	0.006	0.010
FAC4	0.274	0.239	0.135	0.058
FAC5	0.019	0.161	0.454	0.017
FAC6	0.302	0.153	0.027	0.009
FAC7	0.000	-	0.500	0.015
FAC8	0.000	-	0.500	0.018
FAC9	0.084	0.208	0.343	0.006
FAC10	0.342	0.292	0.136	0.014

h^2 ^= heritability estimate (i.e., proportion of phenotype variation attributable to genetics)

h^2 ^SE = standard error of the heritability estimate

R^2 ^= proportion of phenotype variation attributable to the cumulative effects of age, age^2^, and sex

### Factor analysis

**Figure 2:**
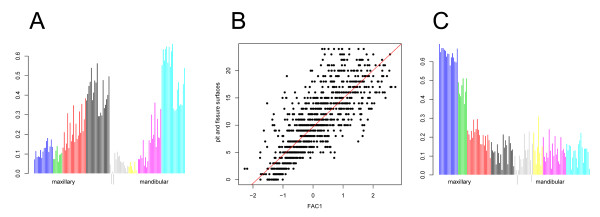
**Factor analysis**. (A) Loadings for FAC1 ordered by tooth type, from left to right: maxillary incisors (blue), canines (green), premolars (red), molars (black), mandibular incisors (gray), canines (yellow), premolars (magenta), molars (cyan). For each tooth, contributions of surfaces are listed in the following order: buccal, distal, lingual, mesial, and occlusal, if applicable. Teeth ordered from left to right: maxillary 8, 9, 7, 10, 6, 11, 5, 12, 4, 13, 3, 14, 2, 15; mandibular 24, 25, 23, 26, 22, 27, 21, 28, 20, 29, 19, 30, 18, 31. (B) Scatter plot of FAC1 vs. pit and fissure surface caries. (C) Loadings for FAC2 in the same order.

FA was also performed on the surface-level data to identify latent patterns of dental decay (Table 2). 10 factors were extracted which cumulatively explained 44.7% of the variability of the data. FAC1 was primarily due to the contributions of molar surfaces, and to a lesser degree, premolar surfaces (see loadings, Figure 2A). FAC1 was moderately correlated with DMFS index (r = 0.593; p-value < 10^-250^), and strongly correlated with PFS (r = 0.815, p-value < 10^-250^; Figure 2B). Loadings showed that maxillary incisor surfaces, and to a lesser degree, maxillary canine surfaces, contribute to FAC2 (Figure 2C). FAC2 was moderately correlated with SMS (r = 0.523; p-value < 10^-250^) and DMFS index (r = 0.453; p-value < 10^-250^). See Additional file 1 for loadings of all other factors. In general, most FACs showed low correlations with PCs, indicating that the two methods extracted different patterns from the data. Compared with the PCs, which represented contributions from many teeth, a number of FACs primarily represented contributions of individual teeth (e.g., tooth 20 for FAC7, tooth 29 for FAC8, tooth 13 for FAC10).

The heritability estimates of FACs 1-10 are also shown in Table 3. FAC3 and FAC6 were strongly heritable (h^2 ^= 65.3 and 30.2%; p-value = 0.006 and 0.027, respectively), whereas all other FACs were not heritable. These results echo the PCA results, showing that some caries patterns are due to genetic etiologies, whereas others are not. Significance levels for heritability estimates did not meet Bonferroni adjustment (for 20 models, requiring p-values < 0.0025 for family-wise significance); although, correct adjustment for multiple testing is not clear given the prior significant heritability of DMFS, PFS, and SMS indices reported for this sample [6,36].

## Discussion

We used two related methods of extracting caries patterns in the permanent dentition from surface-level caries data. PCA yielded many moderate-to-weak patterns, possibly indicating a high degree of noise or sporadic (non-patterned) occurrence of dental caries. Moreover, PCs 1-3 closely recaptured the DMFT, PFS, and SMS indices, an observation that suggests these *a priori *caries phenotypes may reflect the predominant patterns of decay in the permanent dentition, although cumulatively they account for only 37% of the variability. Some PCs were heritable, whereas many were not, which suggests that genetic patterns of decay may be separable from non-genetic patterns. Unlike PCA, FA did not yield factors that clearly recaptured *a priori *phenotypes, with the exception that FAC1 was correlated with PFS. Maxillary incisors contributed heavily to FAC2, which is consistent with previous studies that used multidimensional scaling [24] and cluster analysis [23] to explore caries patterns in the primary dentition and showed maxillary incisors formed the second cluster (after other smooth surfaces). Ten factors were insufficient to explain the variability of the data, cumulatively accounting for approximately 45%.

Like PCA, FA yielded some factors that were highly heritable indicating that certain caries patterns may be due to genetic etiologies while others may be due to non-genetic etiologies. Because the caries patterns presented in this manuscript are more precisely and agnostically defined than *a priori *phenotypes, we conservatively conclude that specific patterns represented by FAC3 and FAC6 are heritable, rather than generalizing to broader surface categories such as SMS. Interestingly, the strongest genetic contribution identified was for FAC3, which was 65.3% heritable (compared to 41.8% for D1MFS index) which suggests that FAC3 may be a better phenotype for gene discovery than *a priori *caries phenotypes. A similar conclusion can be made for PC7 (50.3% heritable). These results are generally consistent with a previous study comparing PCA and FA that showed FA may better capture underlying genetic signals from correlated phenotype measurements (although both methods perform quite similarly) [35]. Non-heritable PCs and FACs, presumably due to effects of non-genetic risk factors, may be preferred phenotypes for future epidemiological studies of environmental risk factors for dental caries.

The severity of caries significantly increased with age (or age^2^) for most patterns (results not shown). Heritability estimates were calculated while simultaneously modeling age, age^2^, and sex, although very similar heritability estimates were obtained in unadjusted models for all patterns except PC1 which exhibited decreased heritability when covariates were omitted (results not shown). These results are sensible given that altogether, age, age^2 ^and sex accounted for about 10% of variance in PC1, but very little variance for the other PCs and FACs.

One of the challenges of using agnostic methods such as PCA and FA to identify underlying patterns of dental decay (devoid of *a priori *surface classifications) is in interpreting the findings. While some patterns, such as PC1 (defined by near-uniform loadings across most tooth surfaces and therefore representing global extent of decay), and FACs 7, 8 and 10 (each defined by contributions of a single pre-molar), are readily interpretable, other PCs and FACs may be difficult to relate back to the original variables. Moreover, there is no clear method of distinguishing biologically relevant patterns attributable to distinct risk factors from sporadic patterns due to noise. Sensitivity analysis showed that patterns represented by PCs 1-9 and FACs 1-6 were stable, whereas PC10 and FACs 7-10 were moderately stable. The overall stability lends credence to the notion that PCs and FACs considered in this study are not due to chance alone.

This study benefits from the large sample of related individuals with detailed surface-level caries assessment, which facilitated caries pattern extraction and heritability estimation. An additional strength of the analysis was using two different but related methods of extracting caries patterns from the data, which, most importantly, did not use *a priori *pattern definitions.

Despite these strengths, several limitations of this study warrant discussion, including inherent limitations to assigning tooth surfaces as carious or not. First, caries assessment by visual inspection, though suitable for obtaining data on large numbers of individuals and of sufficient quality for research purposes, may under-represent the true level of disease. Moreover, teeth missing due to decay, for which all surfaces count as carious, and approximal lesions which are often treated by two-surface restorations (leading to filled occlusal surfaces despite absence of decay) may cause caries assessment errors. Likewise, the quality of caries assessment may not be uniform across surfaces of the permanent dentition, which may have caused additional "noise" in the caries measurement. Lastly, prophylactic restorations may inflate caries assessment. These limitations are unavoidable for cross-sectional (i.e., single time point) study designs of dental caries. However, appropriate modeling techniques, such as methods of pattern extraction including PCA and FA, may aid in overcoming theses limitations of the caries assessment.

## Conclusions

To our knowledge, this study is the first exploration of caries patterns in the permanent dentition in adults without relying on *a priori *assumptions or surface classifications. Overall, this study demonstrates the utility of methods for extracting caries patterns from surface-level data and reinforces the complexity of dental caries etiology. Because risk factors that manifest as specific decay patterns may otherwise go unobserved with respect to global or other *a priori *caries phenotypes, the use of patterns as novel phenotypes may assist in understanding the multifactorial nature of dental caries. This study is one of few but much needed efforts to use decay patterns to define new phenotypes for studying dental caries.

## Abbreviations

DMFS: Decay missing filled surfaces; DMFT: Decay missing filled teeth; PCA: Principal components analysis; FA: Factor analysis; PC: Principal components score; FAC: Factor score; PFS: Pit and fissure surface caries; SMS: Smooth surface caries.

## Competing interests

The authors declare that they have no competing interests.

## Authors' contributions

JRS conceived and designed this study; RJW, RC, DWM, and MLM conceived and designed the COHRA initiative; JRS analyzed the data; JRS, EF, WX, KT, DEW, RSD, and MLM managed, cleaned and quality checked the data; JRS, EF, WX, KT, DEW, RSD, DEP, SW, RJW, RC, DWM, and MLM interpreted the results; JRS wrote the manuscript; JRS, EF, XW, KT, DEW, RSD, DEP, SW, RJW, RC, DWM, and MLM read, revised and approved the manuscript.

## Pre-publication history

The pre-publication history for this paper can be accessed here:

http://www.biomedcentral.com/1472-6831/12/7/prepub

## Supplementary Material

Additional file 1 **Provides graphs of loadings for PCs 1-10 and FACs 1-10**. Click here for file
