# Supplementary material for: Heritable patterns of tooth decay in the permanent dentition: principal components and factor analyses
Source: BMC Oral Health. 2012 Mar 9;12:7. doi: 10.1186/1472-6831-12-7 (PMC3328249; doi:10.1186/1472-6831-12-7)
Supplement: Additional file 1 — Provides graphs of loadings for PCs 1-10 and FACs 1-10. [file 1472-6831-12-7-S1.PDF]

Supplemental Material

Heritable patterns of tooth decay in the permanent dentition: Principal components and factor analyses

John R. Shaffer[1, 2], Eleanor Feingold[1, 2, 4], Xiaojing Wang[2, 3, 5], Karen T.Cuenco[2, 3, 5, 6], Daniel E. Weeks[1, 4], Rebecca S. DeSensi[2, 3, 5], Deborah E. Polk[2, 6, 7], Steve Wendell[2, 3, 5], Robert J. Weyant[2, 6], Richard Crout[2, 8], Daniel W. McNeil[9], Mary L. Marazita[1, 2, 3, 5, 10]

[1] Department of Human Genetics, Graduate School of Public Health, University of Pittsburgh, Pittsburgh, PA, 15261

[2] Center for Oral Health Research in Appalachia, University of Pittsburgh, Pittsburgh, PA, 15261 and West Virginia University, Morgantown, WV, 26506

[3] Center for Craniofacial and Dental Genetics, School of Dental Medicine, University of Pittsburgh, Pittsburgh, PA, 15219

[4] Department of Biostatistics, Graduate School of Public Health, University of Pittsburgh, Pittsburgh, PA, 15261

[5] Department of Oral Biology, School of Dental Medicine, University of Pittsburgh, Pittsburgh, PA, 15261

[6] Department of Dental Public Health and Information Management, University of Pittsburgh, School of Dental Medicine, Pittsburgh, PA,15261

[7] Department of Behavioral and Community Health Sciences, Graduate School of Public Health, University of Pittsburgh, Pittsburgh, PA, 15261

[8] Department of Periodontics, West Virginia University School of Dentistry, Morgantown, WV, 26506

[9] Dental Practice and Rural Health, West Virginia University of Dentistry, Morgantown, WV, 26506

[10] Clinical and Translational Science Institute, and Department of Psychiatry, School of Medicine, University of Pittsburgh, Pittsburgh, PA.

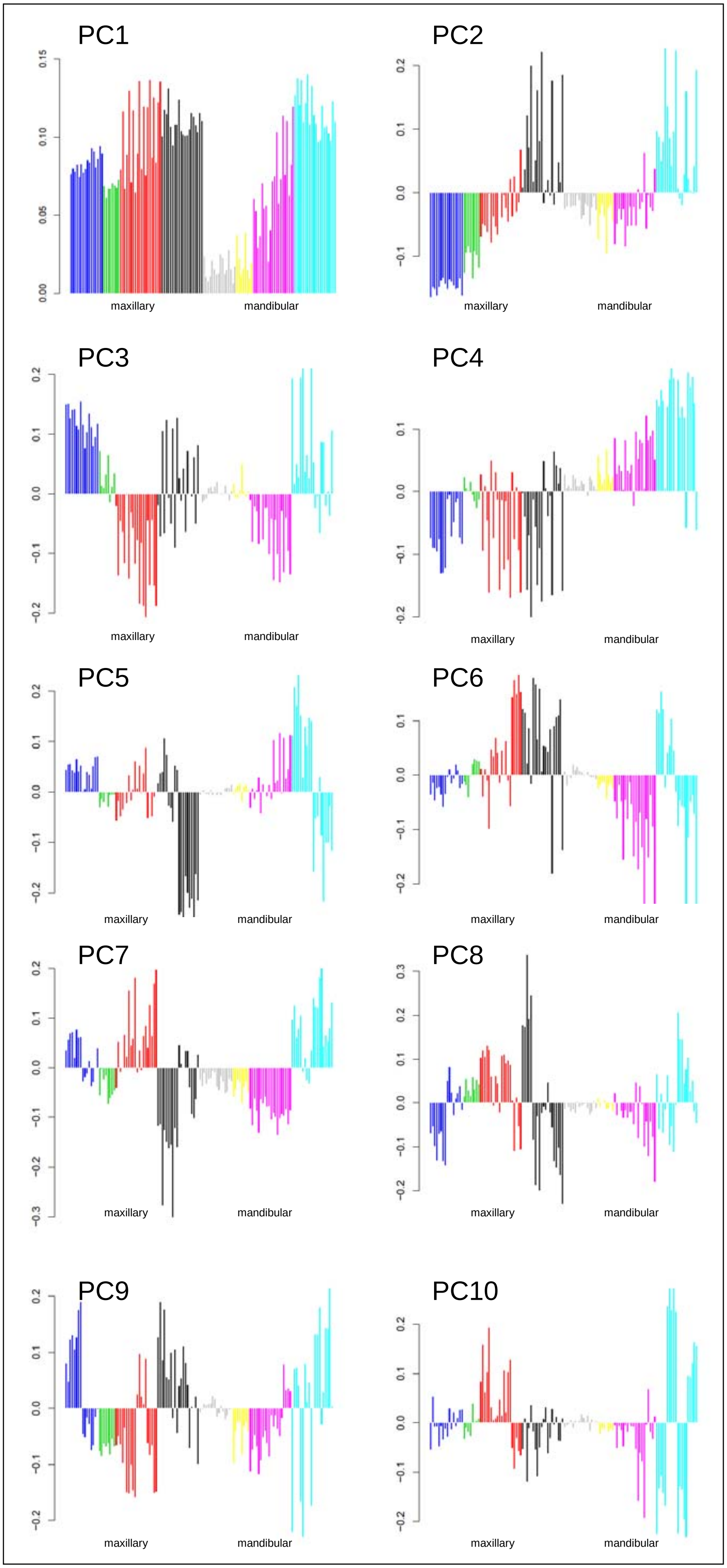

Figure S1: Loadings for PCs 1-10 ordered by tooth type, from left to right: maxillary incisors (blue), canines (green), premolars (red), molars (black), mandibular incisors (gray), canines (yellow), premolars (magenta), molars (cyan). For each tooth, contributions of surfaces are listed in the following order: buccal, distal, lingual, mesial, and occlusal, if applicable. Teeth ordered from left to right: maxillary 8, 9, 7, 10, 6, 11, 5, 12, 4, 13, 3, 14, 2, 15; mandibular 24, 25, 23, 26, 22, 27, 21, 28, 20, 29, 19, 30, 18, 31.

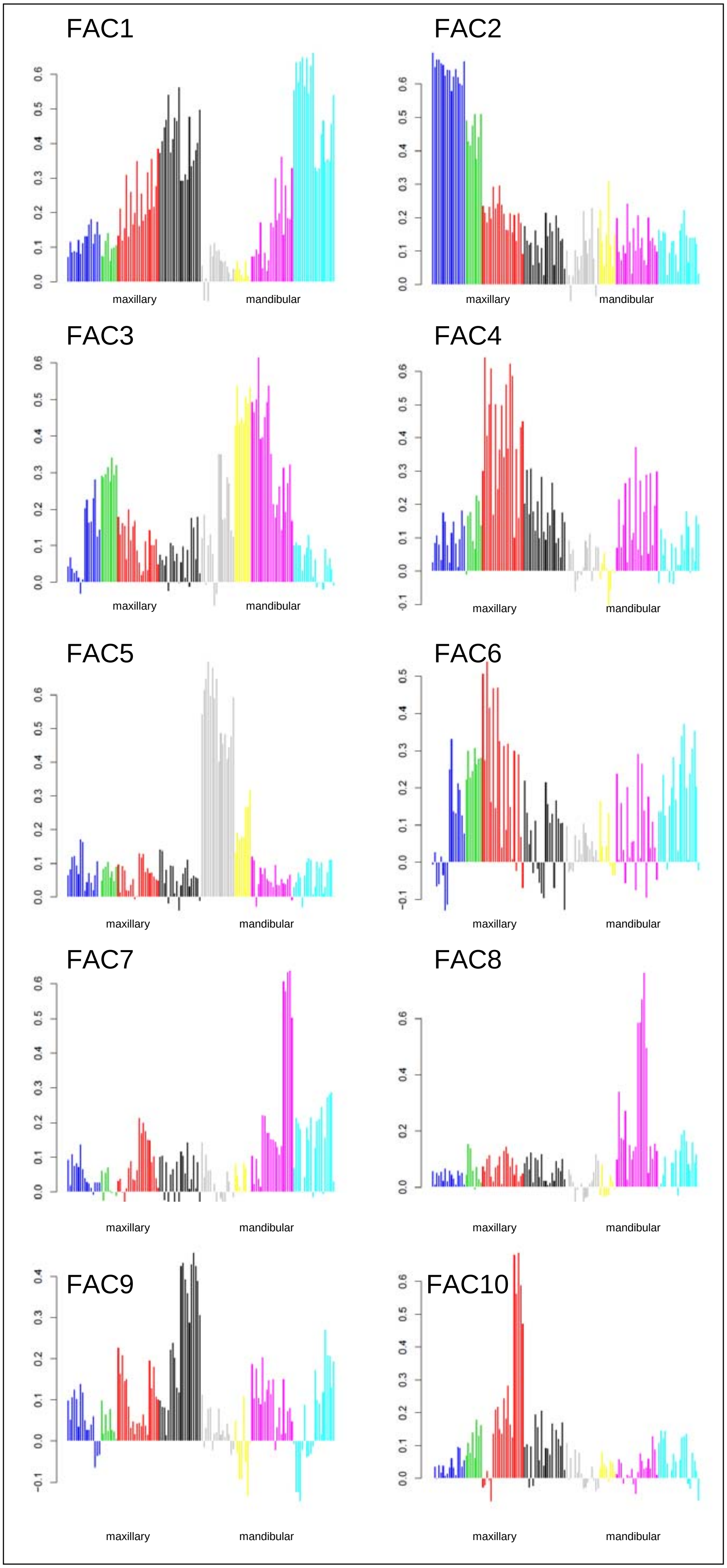

Figure S2: Loadings for FACs 1-10 ordered by tooth type, from left to right: maxillary incisors (blue), canines (green), premolars (red), molars (black), mandibular incisors (gray), canines (yellow), premolars (magenta), molars (cyan). For each tooth, contributions of surfaces are listed in the following order: buccal, distal, lingual, mesial, and occlusal, if applicable. Teeth ordered from left to right: maxillary 8, 9, 7, 10, 6, 11, 5, 12, 4, 13, 3, 14, 2, 15; mandibular 24, 25, 23, 26, 22, 27, 21, 28, 20, 29, 19, 30, 18, 31.
